# Role of NF-kB RelB in Aryl Hydrocarbon Receptor-Mediated Ligand Specific Effects

**DOI:** 10.3390/ijms20112652

**Published:** 2019-05-30

**Authors:** Yasuhiro Ishihara, Sarah Y. Kado, Christiane Hoeper, Shelly Harel, Christoph F. A. Vogel

**Affiliations:** 1 Center for Health and the Environment, University of California, One Shields Avenue, Davis, CA 95616, USA; yaishihara@ucdavis.edu (Y.I.); sykado@ucdavis.edu (S.Y.K.); choeper@ucdavis.edu (C.H.); sharel@ucdavis.edu (S.H.); 2 Graduate School of Integrated Sciences for Life, Hiroshima University, Hiroshima 739-8521, Japan; 3 Department of Environmental Toxicology, University of California, One Shields Avenue, Davis, CA 95616, USA

**Keywords:** AhR, cytokines, DC, IDO, NF-κB RelB, TCDD

## Abstract

Here, we investigate the role of RelB in the regulation of genes which were identified to be induced in an aryl hydrocarbon receptor (AhR)-dependent manner and critically involved in regulation of immune responses. We analyzed the expression of genes of the AhR gene battery, cytokines, and immune regulatory enzymes in bone marrow-derived macrophages (BMM) and thymus of B6 wildtype (wt) mice and RelB knockout (RelB^−/−^) mice after treatment with various AhR ligands. The 2,3,7,8-tetrachlorodibenzo-p-dioxin (TCDD)-induced expression of indoleamine 2,3-dioxygenase 1 (IDO1) and IDO2 was significantly repressed in thymus of RelB^−/−^ mice but not in BMM derived from RelB^−/−^ mice. Interestingly, the induced and basal expression of the cytokines interleukin (IL)-17A, IL-22, and CCL20 required the functional expression of RelB. The RelB-dependent expression of CCL20 was induced by the AhR ligands TCDD and 6-formylindolo[3,2-b]carbazole (FICZ), whereas indole-3-carbinol (I3C) suppressed CCL20 in lipopolysaccharide (LPS)-activated wt BMM. The LPS-induced expression of IL-6 and IL-10 was enhanced by TCDD and FICZ, whereas I3C significantly suppressed these cytokines in BMM. The exposure to FICZ led to higher increases of IL-17A and IL-22 mRNA compared to the effect of TCDD or I3C in thymus of wt mice. On the other hand, TCDD was the strongest inducer of CYP1A1, AhR Repressor (AhRR), and IDO2. In summary, these findings provide evidence for the important role of RelB in the transcriptional regulation of cytokines and enzymes induced by AhR ligands.

## 1. Introduction

The mechanism of transcriptional activation of CYP1A1 and other genes of the AhR gene battery has been well studied. The AhR is a member of the helix-loop-helix, Per-Arnt-Sim (PAS) protein family which is located in the cytoplasm in complex with chaperons including the heat shock protein 90 (HSP90) [1,2]. The ligand-activated AhR translocates into the nucleus to form a heterodimer with the aryl hydrocarbon receptor nuclear translocator (ARNT). The activated AhR/ARNT complex binds on consensus dioxin-responsive elements (DREs) located in the regulatory region of genes of the AhR gene battery especially genes encoding phase I and phase II enzymes (e.g., CYP1a1, CYP1a2, and Ugt1a6), which are responsible for metabolizing xenobiotics [3]. The flanking regions of the DRE core sequence play a critical role and may decide if the core sequence would serve as a functional AhR/ARNT-binding site of AhR-regulated genes [4]. While it is clear that the canonical AhR signaling pathway is a fundamental mechanism to mediate biological and toxicological effects of AhR ligands, recent studies show that the AhR may interact with other signaling pathways [5]. For instance, the induction of the plasminogen activator inhibitor-1 (PAI-1) by AhR ligands requires the interaction of AhR with the Krüppel-like factor 6 (KLF6) and binding to a novel non-consensus DRE (nc-DRE) located in the promoter region of PAI-1 as shown by Wilson and his co-workers [6]. The AhR plays also a critical role in T-cell differentiation which involves the interaction with STAT1 and STAT5 [7]. Recent studies show that the AhR is a key regulator for development and function of interleukin (IL)-22-secreting innate lymphoid cells (ILCs) in the lamina propria [8,9]. Furthermore, Francisco Quintana’s group showed that the production of IL-22 in CD4+ T-cells involves the interaction of AhR with RAR-related orphane receptor gamma t (RORγt) [10]. Interaction of AhR with cMAF in dendritic cells (DCs) has been shown to be important for the regulation of IL-10 and IL-21, which resulted in the generation of type 1 regulatory T (Tr1) cells [11,12]. The expression of certain cytokines and chemokines has been found to be modified through activation of AhR and interaction with the transcription factor NF-κB [13,14]. Previously, we have shown that the induction of chemokines such as IL-8 involves the interaction of AhR with the NF-kB subunit RelB [13,15]. The AhR signaling pathway may also include ligand-independent activation of AhR via intracellular signal induction through the second messenger cyclic adenosine monophosphate (cAMP) and protein kinase A (PKA) [16], which inspired us to propose a non-canonical AhR pathway [13]. The innate immune functions and differentiation of DC are critically regulated through NF-κB RelB [17]. More recent reports have shown that the AhR signaling pathway may modify differentiation and function of macrophages and DCs [18,19,20]. Reports revealed that the AhR, like NF-κB, is involved in many different aspects of the fine-tuned mechanisms and functions of the immune system [21,22,23]. Deficiency or alteration of the activity of AhR and NF-κB RelB have been shown to affect development of T lymphocytes and DCs associated with immunodeficiency and autoimmunity [17,19,20,24,25]. Since both signaling pathways, AhR and NF-κB, are interacting and regulating the function of innate immune cells, it is important to examine the role of RelB in responses mediated by AhR ligands. The current study investigates the function of RelB as an important regulator of the mRNA expression of cytokines and immune regulatory enzymes in thymus and bone marrow-derived macrophages (BMM) mediated by AhR ligands.

## 2. Results and Discussion

### 2.1. Effects of AhR Ligands on the Expression of Genes of the AhR Gene Battery, Cytokines, and Immune Regulatory Enzymes in Bone Marrow-Derived Macrophages (BMM) Derived from wt and RelB^−/−^ Mice

**Figure 1:**
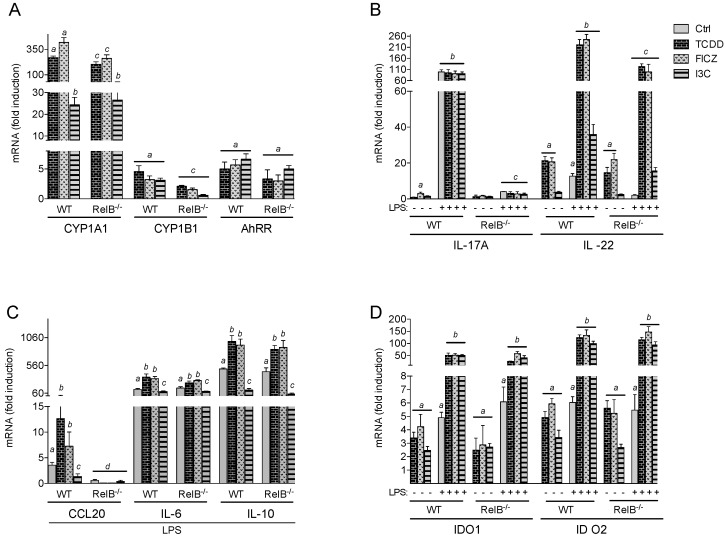
Effects of AhR ligands on CYP1A1, CYP1B1, AhRR, cytokines, and IDO expression in BMM. The mRNA expression of (**A**) CYP1A1, CYP1B1, and AhRR induced by AhR ligands in BMM is shown. BMM derived from wt or RelB^−/−^ mice were treated with 1 nM TCDD, 100 nM FICZ, or 50 μM I3C for 24 h. Results from real-time PCR are presented as mean ± SEM and the y-axis represents mRNA expression level as fold increase above control. ^a^ significantly higher than BMM control, *p* < 0.05; ^b^ significantly lower than TCDD- or FICZ-treated wt or RelB^−/−^ BMM, *p* < 0.05; ^c^ significantly lower than wt BMM, *p* < 0.05. (**B**) Expression of IL-17A and IL-22 in BMM derived from wt or RelB^−/−^ mice treated with 1 nM TCDD, 100 nM FICZ, or 50 μM I3C for 24h in the absence or presence of LPS (50 ng/mL). ^a^ significantly higher than wt BMM control, *p* < 0.05; ^b^ significantly higher than FICZ- or LPS-treated wt BMM, *p* < 0.05; ^c^ significantly lower than wt BMM, *p* < 0.05. (**C**) Expression of CCL20, IL-6, and IL-10 in BMM derived from wt or RelB^−/−^ mice treated with 1 nM TCDD, 100 nM FICZ, or 50 μM I3C for 24 h in the presence of LPS (50 ng/mL). ^a^ significantly higher than BMM control, *p* < 0.05; ^b^ significantly higher than LPS-treated BMM, *p* < 0.05; ^c^ significantly lower than LPS, TCDD, or FICZ-treated BMM, *p* < 0.05; ^d^ significantly lower than wt BMM, *p* < 0.05. (**D**) Expression of IDO1 and IDO2 in BMM derived from wt or RelB^−/−^ mice treated with 1 nM TCDD, 100 nM FICZ, or 50 μM I3C for 24 h in the presence or absence of LPS (50 ng/mL). ^a^ significantly higher than BMM control, *p* < 0.05; ^b^ significantly higher than LPS-treated BMM, *p* < 0.05.

BMM derived from wt and RelB^−/−^ mice were differentiated ex vivo and used to analyze the effect of TCDD, FICZ, and I3C on the mRNA expression of CYP1A1, CYP1B1, and AhRR. Treatment of wt BMM with 100 nM FICZ led to a clear induction of CYP1A1 (418-fold) followed by a 269-fold increase induced by TCDD (1 nM) in wt BMM (Figure 1A). Treatment with 50 µM I3C led to a moderate increase of CYP1A1 by 24-fold above control after 24 h treatment. The induction of CYP1A1 by TCDD and FICZ was significantly lower in RelB^−/−^ BMM by 38% and 24%, respectively. The effect of I3C on CYP1A1 expression did not significantly change between wt and RelB^−/−^ BMM. The expression of CYP1B1 increased by 4.5-fold after treatment with TCDD and led to a 3-fold increase by FICZ and I3C in wt BMM. The expression of CYP1B1 was significantly lower in AhR ligand-treated RelB^−/−^ BMM. The induction of AhRR mRNA expression by 5- to 6-fold via AhR ligands TCDD, FICZ, and I3C in wt BMM did not significantly change in RelB^−/−^ BMM. These results indicate that the induction of CYP1A1 is affected in cells lacking RelB in an AhR ligand-dependent manner. The results also show that RelB plays a role in mediating the induction of CYP1B1 but does not affect the expression of AhRR by AhR ligands.

Furthermore, we tested if the deficiency of RelB may change the effect of AhR ligands on the expression of genes which are critically involved in the regulation of immune responses. Figure 1B shows the effect of AhR ligands on the expression of IL-17A and IL-22 in the absence or presence of LPS. The results show that FICZ, but not TCDD or I3C, led to a 3-fold increase of IL-17A mRNA in wt BMM. The mRNA expression of IL-17A was about 90-fold upregulated by LPS above control in wt BMM, which was not significantly affected in the presence of AhR ligands. In contrast, the mRNA expression of IL-17A was drastically repressed in RelB^−/−^ BMM and LPS or AhR ligands had no effect on IL-17A in RelB^−/−^ BMM. The expression of IL-22 was about 20-fold increased by TCDD and FICZ and 3.2-fold by I3C in wt BMM. LPS alone led to 12.6-fold increase of IL-22 in wt BMM, which was significantly increased when AhR was activated by TCDD (220-fold), FICZ (245-fold) or I3C (36-fold). While the induction of IL-22 in RelB^−/−^ BMM by AhR ligands was not significantly different from wt BMM, the effect of LPS was significantly repressed in RelB^−/−^ BMM. The results clearly indicate that the expression of IL-17A induced by FICZ or LPS requires RelB and that the LPS- but not AhR ligand-mediated induction of IL-22 depends on RelB. The expression of IL-22 and IL-17A has been primarily associated with lymphoid cells including activated T-cells expressing high levels of AhR [9,26,27]. In the current study, we analyzed the mRNA expression of IL-17A and IL-22 in BMM, but we did not determine if the induced expression of mRNA would result in a detectable level or biological activity of the corresponding proteins. The stimulatory effect of AhR ligands on IL-22 expression in LPS-activated wt BMM confirms previous studies in bone marrow-derived DCs and CD4+ T-cells [19,28].

The chemokine CCL20 plays a critical role in the chemoattraction of CCR6 expressing cells such as T-cells. A previous study has shown that activation of AhR synergistically induced the LPS-stimulated expression of CCL20 in primary peritoneal macrophages isolated from B6 mice [29]. In the current study, we confirmed that TCDD and FICZ significantly increased the expression of CCL20 in LPS-activated wt BMM (Figure 1C). In contrast, I3C significantly suppressed the LPS-induced CCL20 expression. Similar to those of IL-17A, the stimulatory effects of LPS and AhR ligands on CCL20 expression were completely RelB-dependent.

Treatment with AhR ligands alone had no effect on the expression of CCL20, IL-6, or IL-10 in the absence of LPS (data not shown). However, the LPS-induced expression of IL-6 and IL-10 was further increased after treatment with TCDD or FICZ. In contrast, I3C significantly repressed the mRNA levels of IL-6 and IL-10 induced by LPS in wt and RelB^−/−^ BMM. The results clearly indicate that the AhR- as well as Toll-like receptor (TLR)-stimulated expression of CCL20 depends on the presence of RelB. In contrast to TCDD and FICZ, which both stimulated the LPS-induced expression of CCL20, IL-6, and IL-10, the dietary AhR ligand I3C repressed the LPS-induced expression of the cytokines in a RelB-independent manner.

Furthermore, we analyzed the expression of the immune regulatory enzymes indoleamine-2,3-dioxygenase 1 (IDO1) and IDO2 in BMM. As shown earlier in U937-derived DCs and bone marrow-derived DC (BMDC) from mice [19,20,30], the expression of IDO1 and IDO2 was significantly increased after ligand-dependent activation of AhR in wt BMM (Figure 1D). As shown in human monocyte-derived DC (moDC) [31], the activation of AhR synergistically increased the LPS-induced expression of IDO1 and IDO2. The expression of IDO1 and IDO2 induced by LPS and AhR ligands did not significantly change between wt and RelB^−/−^ BMM.

### 2.2. Effects of TCDD on the Expression of Genes of the AhR Gene Battery, Cytokines, and Immune Regulatory Enzymes in Thymus of wt and RelB^−/−^ Mice

**Figure 2:**
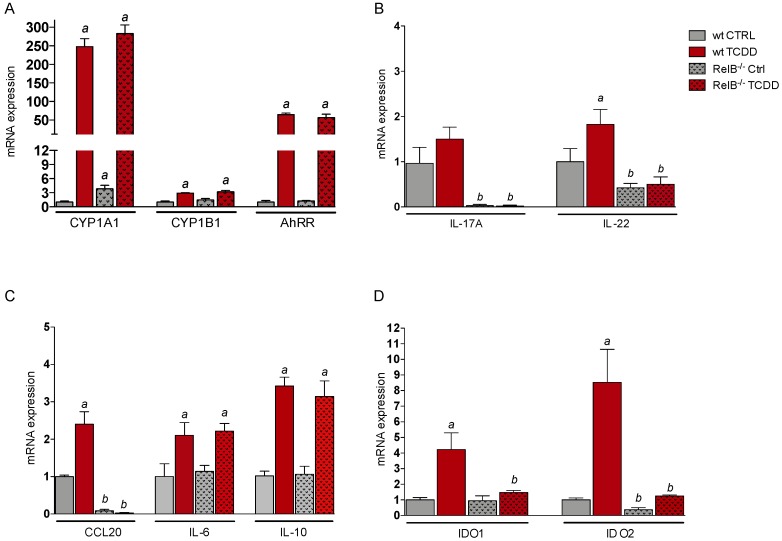
Effects of TCDD on CYP1A1, CYP1B1, AhRR, cytokines, and IDO expression in thymus of wt and RelB^−/−^ mice. Male wt (*n* = 5) and RelB^−/−^ mice (*n* = 5) were treated with 15 μg/kg TCDD for 24 h and expression of (**A**) CYP1A1, CYP1B1, and AhRR induced by TCDD is shown. ^a^ significantly higher than wt control, *p* < 0.05. (**B**) Expression of IL-17A and IL-22 induced by TCDD is shown. ^a^ significantly higher than wt control, *p* < 0.05. ^b^ significantly lower than wt control, *p* < 0.05. (**C**) Expression of CCL20, IL-6, and IL-10 induced by TCDD is shown. ^a^ significantly higher than wt control, *p* < 0.05. ^b^ significantly lower than wt control, *p* < 0.05. (**D**) Expression of IDO1 and IDO2 induced by TCDD is shown. ^a^ significantly higher than wt control, *p* < 0.05. ^b^ significantly lower than wt control or TCDD-treated wt, *p* < 0.05. ^a^ significantly higher than wt control, *p* < 0.05. ^b^ significantly lower than wt control or TCDD-treated wt, *p* < 0.05.

B6 wt and RelB^−/−^ mice were treated with 15 µg/kg TCDD via intraperitoneal (i.p.) injection for 24 h and gene expression was determined in thymus using qPCR. TCDD induced the expression of CYP1A1 by about 250-fold in thymus of wt B6 mice (Figure 2A). The TCDD-induced expression of CYP1A1 in thymus of RelB^−/−^ mice was not significantly different from wt mice; however, the constitutive expression of CYP1A1 was 4-fold higher in thymus of control RelB^−/−^ mice compared to that in wt mice. TCDD induced CYP1B1 mRNA expression 3-fold in wt and RelB^−/−^ mice and led to 65- and 56-fold increases of the AhRR mRNA level in thymus of wt and RelB^−/−^ mice, respectively.

The expression of IL-17A in thymus of wt mice was slightly elevated by TCDD (1.5-fold) above control, which was not statistically significant (Figure 2B). As observed in BMM, the expression of IL-17A was drastically reduced in thymus of RelB-deficient mice. TCDD induced the expression of IL-22 (1.8-fold) in thymus of wt mice, whereas the level of IL-22 was significantly lower in control and TCDD-treated RelB^−/−^ mice. The results confirm data from BMM that RelB is required for the expression of IL-17A. Furthermore, stimulation of IL-22 and additional effects of AhR ligands are RelB-dependent.

The CCL20 mRNA level was 2.5-fold increased in thymus of TCDD-treated wt mice compared to that in control animals (Figure 2C). Confirming the results in RelB^−/−^ BMM, we found a substantially lower level of CCL20 in thymus of RelB^−/−^ mice. The expression of IL-6 and IL-10 was increased in thymus of TCDD-treated mice compared to that in control mice. The induction of IL-10 expression mediated by TCDD in thymus of RelB^−/−^ mice tended to be lower, but we did not observe a significant difference between wt and RelB^−/−^ mice (Figure 2C).

Furthermore, we analyzed the expression of the immune regulatory enzymes IDO1 and IDO2 in thymus. We found 4.2- and 8.5-fold increases of IDO1 and IDO2, respectively, in thymus of TCDD-treated wt mice (Figure 2D). In contrast to RelB^−/−^ BMM, there was no effect of TCDD on the expression level of IDO1 or IDO2 in thymus of RelB^−/−^ mice. The basal level of IDO2 was also significantly lower in thymus of control RelB^−/−^ mice compared to that in wt mice. The results suggest that RelB is not an essential factor for the expression of IDO1 or IDO2 in vitro; however, RelB is needed for the development and function of cell types such as macrophages and DCs to express and respond to stimulation of IDO1 and IDO2 by AhR ligands in thymus.

### 2.3. Effects of AhR Ligands on the Expression of Genes of the AhR Gene Battery, Cytokines, and Immune Regulatory Enzymes in Thymus of wt Mice

**Figure 3:**
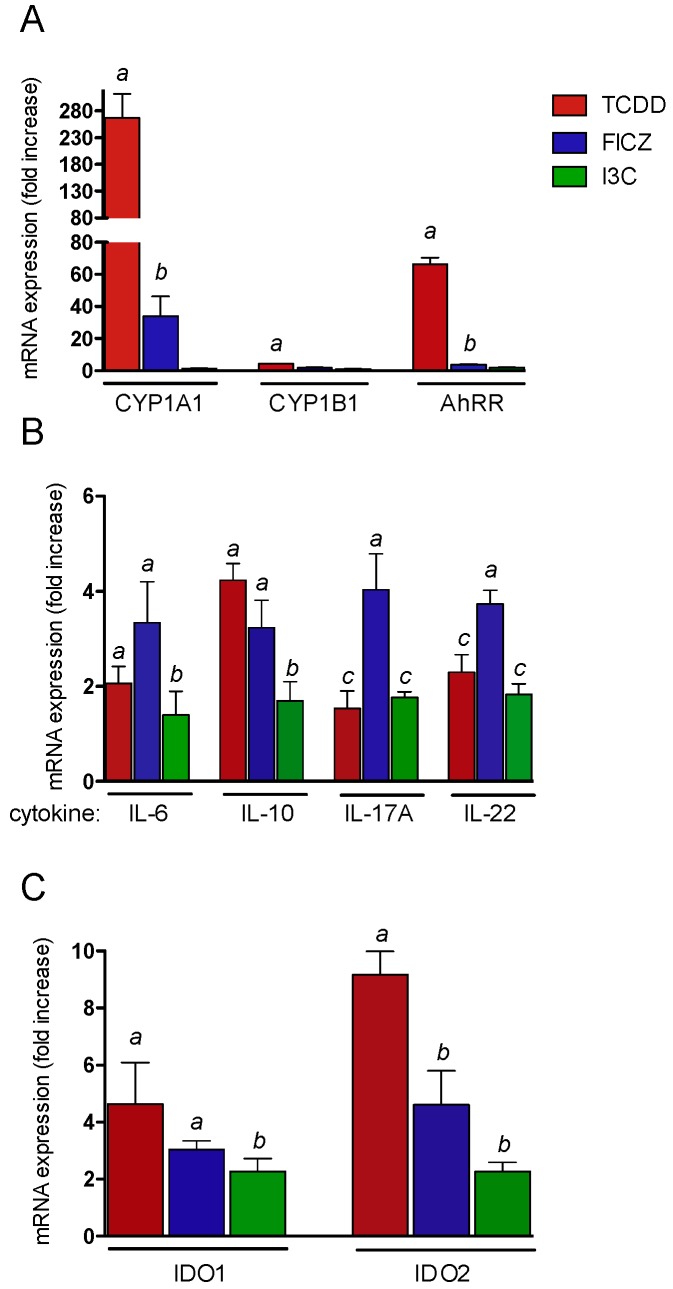
Effects of AhR ligands on CYP1A1, CYP1B1, AhRR, cytokines, and IDO expression in thymus of wt mice. Male wt mice (*n* = 5) were treated with 15 μg/kg TCDD, 150 µg/kg FICZ, and 20 mg/kg I3C via i.p. for 24 h and expression of (**A**) CYP1A1, CYP1B1, and AhRR was analyzed using real-time PCR. ^a^ significantly higher than wt control, *p* < 0.05. ^b^ significantly lower than TCDD, *p* < 0.05. (**B**) Expression of IL-6, IL-10, IL-17A, and IL-22 induced by AhR ligands in thymus of wt mice. ^a^ significantly higher than wt control, *p* < 0.05. ^b^ significantly lower than TCDD or FICZ, *p* < 0.05. ^c^ significantly lower than TCDD or I3C, *p* < 0.05. (**C**) Expression of IDO1 and IDO2 induced by AhR ligands in thymus of wt mice. ^a^ significantly higher than wt control, *p* < 0.05. ^b^ significantly lower than TCDD-treated wt, *p* < 0.05.

Here, we tested the effects of TCDD, FICZ, and I3C on the expression of AhR-regulated genes in thymus of wt mice. B6 wt mice were treated with 15 µg/kg TCDD, 150 µg/kg FICZ, and 20 mg/kg I3C via i.p. for 24 h. RNA was isolated from thymus and the expression of CYP1A1, CYP1B1, and AhRR was analyzed. TCDD led to a clear increase of CYP1A1 by 270-fold above control mice followed by a 34-fold induction after treatment with FICZ (Figure 3A). I3C had no significant effect on the expression of CYP1A1 in thymus of wt mice. The cytokines IL-6 and IL-10 are critical players in differentiation and effector function of T-cells. The expression of IL-6 was 2-fold and 3.3-fold increased in thymus of TCDD- and FICZ-treated mice, respectively. I3C had no significant effect on IL-6 expression in thymus (Figure 3B). TCDD and FICZ induced the expression of IL-10 by 4.2- and 3.2-fold, respectively. The effect of I3C on IL-10 expression was significantly lower compared to that of TCDD or FICZ. The expression of IL-17A was 3.9-fold elevated in thymus of FICZ-treated mice. TCDD and I3C induced IL-17A by 1.8- and 1.9-fold. The expression of IL-22 was 3.7-fold increased by FICZ followed by 2.3- and 1.8-fold inductions after treatment with TCDD and I3C, respectively.

The highest mRNA levels of IDO1 (6.9-fold) and IDO2 (9.2-fold) were found in thymus of TCDD-treated mice (Figure 3C). The effect of I3C was significantly lower on IDO1 compared to that of TCDD and FICZ. The mRNA expression of IDO2 was significantly less induced by FICZ and I3C compared to by TCDD.

## 3. Materials and Methods

### 3.1. Reagents and Antibodies

Dimethylsulfoxide (DMSO) and LPS from *Escherichia coli* O127:B8 were obtained from Sigma-Aldrich (St. Louis, MO, USA). LPS is the major structural component of the outer wall of all Gram-negative bacteria and recognized by TLR4. TCDD (>99% purity) was originally obtained from Dow Chemical Co. (Midland, MI, USA). Other molecular biological reagents were purchased from Qiagen (Valencia, CA, USA) and Roche Clinical Laboratories (Indianapolis, IN, USA).

### 3.2. Animals and Cell Culture

Male wt B6 mice aged 10 weeks were obtained from JAX West, Inc. (Davis, CA, USA) and were euthanized in accordance with a protocol approved by the UC Davis Animal Resources Service. The protocol for animal care and use was approved and completed by the Institutional Animal Care and Use Committee (IACUC) on 10 December 2018 at the University of California Davis (#19671). This project was conducted in accordance with the ILAR guide for the care and use of laboratory animals, and the UC Davis Animal Welfare Assurance on file with the US Public Health Service. RelB^−/−^ mice were kindly provided by Alexander Hoffmann (Department of Microbiology, Immunology and Molecular Genetics at the University of California, Los Angeles). RelB^−/−^ mice were genotyped using the DNA/RNA Shield™ kit (Zymo Research, Irvine, CA, USA). TCDD, FICZ, and I3C were administered via i.p. injection for RNA expression analysis. Primary bone marrow progenitor cells were isolated and differentiated as described previously [32]. Briefly, the femurs were isolated under sterile conditions, and bone marrow cells were extracted via an RPMI medium-loaded syringe. The cells were passed through a 30 μm cell strainer, and the supernatant was centrifuged for 5 min at 800× *g*. The supernatant was decanted, and the pellet was resuspended and cultured in the RPMI medium. Cells were seeded in either 10 cm-diameter or 12-well coated sterile plastic cell culture dishes for culture. For every 78 cm^2^ of culture area, 2 × 10^6^ red cell-depleted marrow cells were plated in 10 mL RPMI medium supplemented with 20 ng/mL recombinant mouse macrophage colony-stimulating factor (rmGM-CSF) and 10% FBS. BMM differentiation was performed in the presence of rmM-CSF (20 ng/mL; Tonbo Biosciences, San Diego, CA, USA) for 7 days. Vehicle, LPS, TCDD, FICZ, or I3C was added to the culture medium at the indicated time points.

### 3.3. RNA Isolation and Quantitative Real-Time RT-PCR

The preparation of total RNA was performed with a Quick RNA isolation kit (Zymo Research) and synthesis of cDNA was conducted as described previously [33]. qPCR was then performed with the LightCycler LS480 (Roche, Indianapolis, IN, USA) using the Fast SYBR Green Master Mix (Applied Biosystems Inc.) according to the manufacturer’s protocol. The primer sequences are presented in Table 1 and mRNA levels were normalized to the level of the housekeeping gene β-actin.

### 3.4. Statistics

The results were analyzed by GraphPad Prism software. All experiments were repeated a minimum of three times, and data are expressed as mean ± SEM. Differences were considered significant at *p* < 0.05. A comparison of two groups was made with an unpaired, two-tailed Student’s *t* test. A comparison of multiple groups was made with analysis of variance (ANOVA) followed by a Dunnett’s or Tukey’s test. A two-way ANOVA was used when data with more than one factor were analyzed.

## 4. Conclusions

In summary, we found that the constitutive, TLR4- and AhR-mediated transcriptional activation of the cytokines IL-17A and CCL20 ex vivo in BMM and in thymus of B6 mice require NF-κB RelB. The IL-22 expression induced by LPS and TCDD was repressed in BMM and thymus of RelB-deficient mice. The induction of IDO1 and IDO2 by TCDD was RelB-dependent in thymus, but not in BMM, suggesting that RelB is required for the development of macrophages and/or DCs producing IDO1 and IDO2 in thymus. The expression of IL-6 and IL-10 in LPS-activated BMM was further increased by AhR ligands TCDD and FICZ; however, the effect of LPS was suppressed by I3C, suggesting that the AhR-mediated induction of IL-6 and IL-10 is ligand-specific. AhR ligand-specific effects were also observed in thymus of B6 wt mice. TCDD was the strongest inducer of CYP1A1, AhRR, IDO1, and IDO2. On the other hand, treatment of B6 wt mice with FICZ led to higher increases of IL-17A and IL-22 in thymus compared to with TCDD or I3C. The data may give additional insight into the underlying mechanisms of differences in the immune responses mediated by different AhR ligands. The identification of AhR ligand-specific effects may also help to develop safe and selective AhR modulators (SAhRMs) for successful clinical applications.

## Figures and Tables

**Table 1:** Primers used to amplify mRNAs via quantitative real-time PCR based on published GenBank sequences for mice.

Gene	Forward (5′-3′)	Reverse (5′-3′)
*β-actin*	AGCCATGTACGTAGCCATCC	CTCTCAGCTGTGGTGGTGAA
*AhRR*	TGGACAAGCTTTCTGTCCTG	CGAAGCCATTGAGAGACTCC
*CYP1A1*	GGCCACTTTGACCCTTACAA	CAGGTAACGGAGGACAGGAA
*CYP1B1*	TTCTCCAGCTTTTTGCCTGT	TAATGAAGCCGTCCTTGTCC
*IL-6*	AGTTGCCTTCTTGGGACTGA	TCCACGATTTCCCAGAGAAC
*IL-10*	CCAAGCCTTATCGGAAATGA	TTTTCACAGGGGAGAAATCG
*IL-17A*	AGGCCCTCAGACTACCTCAACCGTT	TGGTCCAGCTTTCCCTCCGCATT
*IL-22*	TTTCCTGACCAAACTCAGCA	TCTGGATGTTCTGGTCGTCA
*IDO-1*	GGCTAGAAATCTGCCTGTGC	AGAGCTCGCAGTAGGGAACA
*IDO-2*	GCTATCACCATGGGATTCGT	AGAGATCTTGGCAGCACCTT
